# Pensions, consumption and health: evidence from rural South Africa

**DOI:** 10.1186/s12889-020-09666-6

**Published:** 2020-10-20

**Authors:** Peter Lloyd-Sherlock, Sutapa Agrawal, Francesc Xavier Gómez-Olivé

**Affiliations:** 1 School of International Development, University of East Anglia, Norwich, NR4 7QE UK; 2 Public Health Foundation of India, New Delhi, India; 3 MRC/Wits Rural Public Health and Health Transitions Research Unit (Agincourt), School of Public Health, University of the Witwatersrand, South Africa. Harvard Center for Population and Development Studies, Harvard University, Harvard, USA

**Keywords:** Cash transfers; older adults, Consumption, South Africa

## Abstract

**Background:**

Increasing numbers of older people in sub-Saharan Africa are gaining access to pension benefits and it is often claimed that these benefits promote healthy forms of consumption, which contribute to significant improvements in their health status. However, evidence to support these claims is limited.

**Methods:**

The paper uses data for 2701 people aged 60 or over who participated in a population-based study in rural north-eastern South Africa. It analyses effects of receiving a pension on reported food scarcity, body mass index and patterns of consumption.

**Results:**

The paper finds that living in a pension household is associated with a reduced risk of reported food scarcity and with higher levels of consumption of food and drink. The paper does not find that living in a pension household is associated with a higher prevalence of current smoking nor current alcohol consumption. However, the paper still finds that tobacco and alcohol make up over 40% of reported food and drink consumption, and that the correlation between reported food scarcity and body mass index status is imperfect.

**Conclusions:**

The paper does not show significant associations between pension receipt and the selected risk factors. However, the context of prevalent obesity and high shares of household spending allocated to tobacco and alcohol call into question widely-made claims that pensions enhance healthy consumption among older people in low and middle-income countries.

## Background

With reference to sub-Saharan Africa, there is a broad agreement across academic studies and the grey literature that receiving a pension is associated with higher rates of consumption by older people, and it is inferred that this, in turn, is associated with enhanced health and subjective wellbeing [1–6]. Similar claims are made by studies set in other low and middle-income countries, including Peru, Mexico and China [7–9]. Although the evidence supporting this received wisdom is not always robust, there is considerable plausibility to these assertions, especially in settings or for specific groups where income is scarce and food insecurity prevalent. As well as food, additional forms of “healthy” consumption may include medication and personal hygiene products. These arguments have played an important role in persuading governments across the region to extend or to implement new pension schemes for older people. For example, in 2018 the Kenyan government upgraded a limited set of pilot interventions into a universal old age pension scheme, while Uganda set up a new Senior Citizens Grant in 2011.

An underlying assumption of these studies is that increased consumption does not substantially include unhealthy forms of consumption. However, few publications examine the composition of consumption, with the exception of one study in Lesotho, which reports that a significant share was devoted to alcohol, tobacco and unhealthy foodstuffs [10]. More generally, the context of consumption and food scarcity in sub-Saharan Africa is changing rapidly. Notwithstanding substantial pockets of extreme food insecurity, the region as a whole is experiencing a rapid nutritional transition [11]. For example, the prevalence of adult overweight and obesity in urban areas of Cameroon rose from 54 to 83% between 1994 and 2003 [12]. Studies in Ghana and Kenya report that older age is a significant predictor of obesity [13, 14]. Rising rates of overweight/obesity are associated with a rapid increase in the prevalence of NCDs in sub-Saharan Africa, as are growing alcohol and tobacco consumption [15].

In the light of these trends, the persisting narrative that pensions increase consumption and that this in turn enhances the health of older people requires a more rigorous assessment. This paper provides a detailed analysis of the effects of receiving an old age pension on household consumption in rural South Africa. The paper applies a combination of bivariate and multivariate analyses to assess associations between household pension status and a range of outcomes, including self-reported food scarcity, body mass index (BMI) and specific categories of consumption. The analysis is applied to 2015 data from the Health and Aging in Africa: a Longitudinal Study of an INDEPTH Community in South Africa (HAALSI) cohort collected from Agincourt, South Africa.

## Methods

The HAALSI study is a population-based survey that aims to examine and characterize a population of older men and women in rural South Africa with respect to health, well-being, and physical and cognitive function, as well as the social, environmental, and biological factors affecting these domains [16]. Participants were sampled from the existing framework of the Agincourt Health and Socio-Demographic Surveillance System (HDSS) site in Mpumalanga province [17]. The Agincourt HDSS, a rural setting in north eastern South Africa, is overseen by the MRC/Wits Agincourt Research Unit (www.agincourt.co.za). Agincourt is a former “Bantustan” area: territory set aside for the black population during the Apartheid era. Typical of such areas, basic health and education facilities and living standards are poor compared to urban and rural non-Bantustan parts of the country.

Individuals 40 years and older as on July 1, 2014 and permanently living in the study site during the 12 months previous to 2013 census round were found eligible for this study. Using the full 2013 Census data, HAALSI identified a sampling frame of 8974 women and 3901 men aged 40 years and older who met the residence criteria. The target sample size was approximately 5000 for the completed interviews. Assuming an 80% response rate, a total of 6281 women and men were selected for the main household study.

All sampled individuals were visited at home from November 2014 to November 2015. Trained, local fieldworkers collected survey data electronically using Computer Assisted Personal Interviews. Surveys were conducted in the local Shangaan language, with instruments translated from English and back-translated to ensure reliability. Out of the 6281 selected for the study, 391 had moved outside of the study site or were deceased. Out of the remaining 5890 eligible individuals, 5059 (86%) participated in the baseline survey. Overall, the HAALSI interviewees were slightly older than those in the Agincourt HDSS sample frame, and the gender balance was fairly skewed in the Agincourt HDSS sample frame compared to the HAALSI sample or the interviewees (as men were oversampled to obtain gender balance). The data and questionnaires for HAALSI are publically available at http://haalsi.org/.

### Measures

The main exposure variable was membership of pension and non-pension households. Covariates and explanatory variables included age groups (60–69, 70–79, 80+), sex, household wealth (lowest Q1 to highest Q5), education (no formal education, some primary (1–7 years of education), some secondary (8–11 years of education), secondary or more (12+ years of education), food scarcity in the last one year (never, sometimes/often), body mass index (kg/m^2^) (underweight, normal weight, overweight, obese), ever smoked (no, yes), ever drink (no, yes). We have used the standard BMI categories developed by the World Health Organization and the National Heart, Lung, and Blood Institute [18, 19]. These categories are underweight (less than 18.5), normal weight (18.5–24.9), overweight (25.0–29.9), and obese (30.0 or above). The consumption categories include staple food, meat, fruit and vegetables, milk and eggs, spices and oils, beverages and other non-alcoholic drinks such as coffee, tea, juice, water and soft drinks and tobacco and alcoholic beverages (including, beer, wine, spirits).

### Data analysis

All analyses were conducted using STATA statistical software package Version 14 (College Station, Texas). Standard descriptive statistics were calculated for all variables, including means and standard deviations. Bivariate analysis was conducted and differences in categorical variables were tested using χ2 tests. Further, multivariable logistic regression analysis estimated the odds ratios of household pension status on food scarcity, body mass index, current smoking or drinking status adjusted for age and sex of the household members.

## Results

Table 1 presents selected characteristics of the study population, comparing older people living in “pension households” (where at least one member reported receipt of an old age pension) and “non-pension households” (where no members reported receipt of an old age pension). For the total population aged 60 and over, 78.2% lived in pension households. There were significant bivariate associations between pension household membership and female sex, as well as primary education. Table 1 shows that only a minority of older people reported going to sleep hungry (17.2% in non-pension households; 10.6% in pension households). The prevalence of obesity/overweight was considerably higher than underweight, especially for older people in pension households (52.3% versus 5.3%). For the total sample, 24.5% reported that they sometimes consumed alcohol and 6.8% that they used tobacco: there were no significant bivariate associations for pension household membership.

**Table 1:** Sample distribution of households with at least one elderly aged 60+ years receiving no pension and receiving pension, HAALSI 2015

Characteristics	Sample distribution ***n*** = 2701	Non-Pension households ***n*** = 574	Pension households ***n*** = 2127	Chi sq ***p***-value
*Age groups*				0.037
60-69y	1274	49.3	46.6	
70-79y	802	25.4	30.8	
80 + y	625	25.3	22.6	
*Sex*				< 0.0001
Male	1283	55.8	45.3	
Female	1418	44.3	54.7	
*Household wealth*				0.070
Q1(lowest)	553	23.7	19.6	
Q2	545	16.7	21.1	
Q3	539	19.0	20.2	
Q4	550	20.7	20.3	
Q5(highest)	514	19.9	18.8	
*Education*				< 0.0001
No formal education	1600	64.5	58.2	
Some primary (1–7)	860	25.4	33.8	
Some secondary (8–11)	131	3.9	5.2	
Secondary or more (12+)	98	6.3	2.9	
*In the past 1 year how often did you or any household members go to sleep at night hungry because there was not enough food?*				0.003
Never	2087	82.8	89.4	
Sometimes/often	265	17.2	10.6	
In the past 1 year how often did you or any household members go a whole day without eating anything because there was not enough food?				0.001
Never	2105	83.3	90.2	
Sometimes/often	247	16.7	9.8	
*Body Mass Index*				0.022
Underweight	146	5.9	5.3	
Normal weight	929	36.9	33.7	
Overweight	707	26.1	26.2	
Obese	668	19.9	26.1	
Empty/missing	251	11.2	8.8	
Currently smoke				0.086
yes	185	38.7	30.4	
No	2515	61.3	69.6	
Currently drink				0.277
Yes	663	52.5	48.9	
No	2037	47.5	51.1	

Table 2 presents associations between two self-reported items on reported food scarcity and age, sex and pension status. The only significant results are for pension household membership, which is negatively associated with both food scarcity items. Table 3 presents bivariate associations between the same two self-report items on food scarcity and measured BMI. It shows that self-reported food scarcity is associated with lower BMI, although it is noteworthy that over 8.0% of older people measured as obese reported experiencing food scarcity and that 79.8% of those who were underweight did not.

**Table 2:** Multivariate analysis of reported food scarcity

	In the past 1 year how often did you or any household members go to sleep at night hungry because there was not enough food? (0-never; 1-sometimes)	*P* value	In the past 1 year how often did you or any household members go a whole day without eating anything because there was not enough food? (0-never; 1-sometimes)	*P* value
	Adjusted OR[95%CI]		Adjusted OR[95%CI]	
*Household pension status*				
Not receive pension	1.00		1.00	
Receive pension	0.56[0.39–0.82]	0.002	0.53[0.37–0.78]	0.001
*Sex*				
Male	1.00		1.00	
Female	0.84[0.65–1.09]	0.181	0.82[0.64–1.09]	0.175
*Age groups*				
60-69y	1.00		1.00	
70-79y	1.11[0.82–1.51]	0.486	1.11[0.81–1.53]	0.504
80 + y	1.28[0.93–1.77]	0.136	1.32[0.96–1.86]	0.106
*Number of individuals living in the household*				
Living alone	1.00		1.00	
Living with one other	1.26[0.77–2.06]	0.351	1.12[0.68–1.84]	0.657
Living in 3–6 person	0.97[0.65–1.46]	0.894	0.86[0.57–1.30]	0.478
Living in 7+ person household	1.13[0.74–1.73]	0.583	0.99[0.64–1.52]	0.949

**Table 3:** Bivariate associations between reported food scarcity and measured BMI

	In the past 1 year how often did you or any household members go to sleep at night hungry because there was not enough food?		In the past 1 year how often did you or any household members go a whole day without eating anything because there was not enough food?	
Total	Never %	Sometimes %	Never %	Sometimes %
Obese	91.3	8.7	91.6	8.4
Overweight	92.1	7.9	92.3	7.7
Normal	85.3	14.8	87.1	12.9
Underweight	79.8	20.2	79.8	20.2
Missing	90.0	10.0	90.0	10.0
*Chi sq p value*	< 0.0001		< 0.0001	
**Men**				
Obese	88.5	11.5	90.5	9.6
Overweight	92.6	7.5	92.6	7.5
Normal	85.6	14.4	86.7	13.3
Underweight	79.3	20.7	79.3	20.7
Missing	90.1	9.9	90.1	9.9
*Chi sq p value*	0.007		0.008	
**Women**				
Obese	92.2	7.8	92.0	8.0
Overweight	91.8	8.2	92.1	7.9
Normal	84.8	15.3	87.7	12.3
Underweight	81.0	19.1	81.0	19.1
Missing	89.9	10.1	89.9	10.1
*Chi sq p value*	0.002		0.049	

Table 4 presents a multivariate analysis of associations between BMI categories, age, pension household category and sex. This is done in two ways: first, obesity it treated as a single category; then it is combined with overweight. In both models there is a strong positive association with female sex, but not with pension household membership. For both, there are negative associations with older age categories.

**Table 4:** Multivariate analysis of BMI category, age, pension household category and sex

	*Body Mass Index* 0-non-obese (underweight/normal/overweight) 1-obese (obese)
	Adjusted OR[95%CI]
*Household pension status*	
Not receive pension	1.00
Receive pension	1.18[0.93–1.50]
*Sex*	
Male	1.00
Female	3.55[2.91–4.32]
*Age groups*	
60-69y	1.00
70-79y	0.81[0.65–1.00]
80 + y	0.43[0.32–0.56]
	*Body Mass Index* 0-non-obese (underweight/normal) 1-obese (overweight/obese)
	Adjusted OR[95%CI]
*Household pension status*	
Not receive pension	1.00
Receive pension	1.11[0.90–1.36]
*Sex*	
Male	1.00
Female	2.62[2.21–3.09]
*Age groups*	
60-69y	1.00
70-79y	0.76[0.63–0.92]
80 + y	0.48[0.38–0.59]

Table 5 presents bivariate analysis of levels of consumption on different items of food and drink. Tobacco and alcohol accounted for 42.7% and 42.3% of total food and drink consumption in non-pension and pension households respectively. By contrast, the corresponding figures for fruit and vegetables were 3.1 and 4.1%.

**Table 5:** Bivariate analysis of reported household consumption of food and drink

In the last 30 days, how much (mean value in Rand) did permanent household member spend and consume on:	Non-Pension households *n* = 574		Pension households *n* = 2127		Total	
	mean [95%CI]	%	mean [95%CI]	%	mean [95%CI]	%
Staple food	360.0[266.3–453.8]	13.9	318.5[296.0–341.1]	14.0	322.6[300.3–344.8]	13.95
Meat	380.8[346.4–415.1]	14.7	414.2[394.9–433.5]	18.2	410.9[393.2–428.7]	16.45
Fruits and vegetables	80.1[70.2–90.0]	3.1	93.0[74.4–111.7]	4.1	91.9[74.9–108.9]	3.6
Milk and eggs	242.7[55.2–430.8]	9.4	144.1[111.3–177.0]	6.3	153.1[118.7–187.5]	7.85
Spices and oils	268.2[111.1–425.4]	10.3	183.7[151.1–216.3]	8.1	192.3[158.9–225.6]	9.2
Beverages and other non-alcoholic drinks such as coffee, tea, juice, water and soft drinks	154.5[52.6–256.4]	6.0	160.1[127.6–192.6]	7.0	159.6[128.6–190.5]	6.5
Tobacco and alcoholic beverages (including, beer, wine, spirits)	1106.7[339.3–1874.1]	42.7	962.8[717.6–1207.9]	42.3	978.1[744.5–1211.6]	42.5
TOTAL	2592.7		2276.4		2434.6	

Table 6 presents a multivariate analysis of self-reported current smoking and alcohol consumption by age, pension household category and sex. The analysis was conducted separately for both sexes and then for older men and women separately. No positive associations were found for household pension status. For both sexes combined, being aged 70 and over was associated with a lower probability of smoking, compared to being aged 60–69, and being aged 80 and over was associated with a lower probability of smoking, compared to being aged 60–69. The same associations were reported for older men alone. Analysis of older women was limited by the small number of observations. For both sexes combined, there were strong positive associations between male sex and current drinking, but not for current smoking.

**Table 6:** Multivariate analysis of self-reported smoking (current) and alcohol consumption (current) by age, pension household category and sex

Both sexes				
	Currently Smoke 0-no 1- Yes	*P* value	Currently drink 0-no 1- Yes	*P* value
	Adjusted OR[95%CI]		Adjusted OR[95%CI]	
*Household pension status*				
Not receive pension	1.00		1.00	
Receive pension	0.72[0.47–1.11]	0.139	0.87[0.67–1.14]	0.318
*Sex*				
Male	1.00		1.00	
Female	0.53[0.21–1.35]	0.185	0.50[0.40–0.64]	< 0.0001
*Age groups*				
60-69y	1.00		1.00	
70-79y	0.40[0.26–0.61]	< 0.0001	0.89[0.69–1.15]	0.374
80 + y	0.43[0.26–0.74]	0.002	0.65[0.49–0.86]	0.002
Men				
*Household pension status*				
Not receive pension	1.00		1.00	
Receive pension	0.83[0.53–1.29]	0.400	0.91[0.66–1.25]	0.563
*Age groups*				
60-69y	1.00		1.00	
70-79y	0.38[0.25–0.59]	< 0.0001	0.86[0.64–1.16]	0.324
80 + y	0.40[0.23–0.69]	0.001	0.64[0.44–0.92]	0.015
Women				
	Currently Smoke* 0-no 1- Yes	*P* value	Currently drink 0-no 1- Yes	*P* value
	Adjusted OR[95%CI]		Adjusted OR[95%CI]	
*Household pension status*				
Not receive pension	1.00		1.00	
Receive pension	–		0.80[0.50–1.28]	0.356
*Age groups*				
60-69y	1.00		1.00	
70-79y	–		0.97[0.60–1.57]	0.906
80 + y	–		0.66[0.41–1.07]	0.090

*MV analysis cannot be run due to small no of cases

Table 7 shows that only a very small minority of older people had received advice from health professionals about smoking, diet or losing weight. The denominator includes all older people, not just those report that currently smoke or drink, or who are overweight/obese, since it is possible that some may have modified their health risk behaviours as a result of past advice. Despite this, the numbers who report receiving advice are too small to support multivariate analysis.

**Table 7:** Advice received from health professionals

	Sample distribution % [***n*** = 2701]	Non-Pension households ***n*** = 574	Pension households ***n*** = 2127	Chi sq ***p***-value
Has a doctor, nurse or other healthcare worker ever advised you to stop smoking?	19.1 [110]	15.1 [18]	20.1 [92]	0.284
Has a doctor nurse or other health care worker ever told you to change your diet?	12.3 [331]	10.5 [60]	12.7 [271]	0.251
Has a doctor nurse or other health care worker ever advised you to lose weight?	2.9 [78]	2.6 [15]	3.0 [63]	0.796

## Discussion

This study has a number of important limitations. First, it does not refers to a nationally representative population of older adults, and is situated in a specific setting. Second, the total sample size is modest: this both impedes the generation of statistically significant results (as for older women who smoke) and limits the generalisability of the findings. The relatively small number of non-pension households (574) reduces the capacity to study this exposure. Finally, some of the covariates, such as food scarcity, expenditure, smoking and alcohol consumption are based on self-report, and may therefore be affected by reporting biases.

Some of our findings are compatible with claims made in the wider literature. For example, our finding that pension household membership was associated with lower rates of reported food insecurity and higher rates of food and drink consumption matches the findings of quantitative and qualitative studies set in South Africa, Swaziland, Zambia, Tanzania, Ethiopia, Mexico and Peru [1–8]. However, other aspects of the research findings call into question the plausibility of claims that pensions were associated with forms of consumption that are conducive to enhanced health status. Food insecurity was rare, regardless of pension household membership, and the prevalence of obese/overweight was considerably higher than underweight. This matches the findings of other surveys which report a rapid fall in food insecurity in South Africa, as well as with general regional trends, notwithstanding the persistence of significant pockets of extreme food insecurity for some vulnerable groups and settings [11, 20, 21].

Although the bivariate association between BMI categories and reported food scarcity is significant, the correlation is not as strong as might have been expected. There are no other published studies on this relationship for older people, although a study of adolescents in Tanzania found a strong association between reported food insecurity and low BMI [22]. We found that the large majority of underweight older people did not report food scarcity. This may be because low BMI can be a consequence of HIV/AIDS or TB, both of which are prevalent among older adults in the study site [23, 24]. Alternatively, this may reflect under-reporting of food scarcity due to stigma, although this effect has not been observed in other studies. Conversely, over 8% of older people who were obese self-reported food scarcity. This matches findings reported associations between reported food insecurity and obesity in the USA [25].

The prevalence of self-reported current smoking in the study population (6.8%) is low compared to those reported by other studies. For example, a national SAGE 2 survey of South Africans aged 50 and over found that 15.5% were current smokers [26]. Low prevalence may reflect specific aspects of the study sample, including a rural setting and relatively low levels of wealth and education compared to national populations. The reduced risk of smoking at older ages has not been reported in other studies. A survey of African townships reported that being aged 50 and over was associated with higher daily smoking, but does not make age comparisons within the older population [27].

The prevalence of current alcohol consumption (24.5%) was considerably higher than for smoking. It is higher than rates reported by the SAGE 1 survey of South Africans aged 50 and over (15.6%) [28], but lower than rates of regular alcohol consumption reported by a survey of adults in North West Province (mean age 49; 64% of men; 33% of women) [29].

There is no evidence that that living in a pension household was positively associated with current smoking or alcohol consumption by older people. These effects have not been assessed by other studies. However, across all households considerably larger shares of household food and drink consumption were allocated to tobacco and alcohol than to fruit and vegetables. This is in keeping with data on high rates of tobacco and alcohol consumption in countries like South Africa, although studies specifically related to their share of household spending are not available [30]. It is unclear what share of household spending on tobacco and alcohol was due to consumption by older people or by younger family members. There is evidence from other studies that pension income is often shared with other household members, both willingly and coercively [31].

The small numbers of older people who received advice from health professionals about diet, smoking or weight loss are striking given the high levels of reported unhealthy consumption and the prevalence of overweight/obesity in the study population. Similar analysis has not been published elsewhere. The case for health advice is reinforced by the findings of nationally surveys of populations aged 50 and over in South Africa and Ghana in 2014/15 [32]. These show that a substantially larger share of the South African sample (62.4%) reported consuming insufficient fruit and vegetables than did the Ghana sample (48.5%). The same survey found 79.8% of the South African sample reported engaging in insufficient physical activity, compared to 39.4% of the Ghanaian one. These findings are of particular interest, since, compared to South Africa, rates of overweight and obesity are lower in Ghana, health service availability is more limited and, in the absence of extensive pension coverage, average incomes of older people are considerably lower.

Taken together, the findings of this study indicate claims that providing older people with pensions will promote healthier patterns of consumption are increasingly questionable in sub-Saharan African settings. These claims are based on a view of the region which became out-dated as the nutritional transition commenced. Arguably, this nutritional transition is further advanced in South Africa than in less affluent countries in the region. However, data from other studies show a rapid pace of change across the region [11]. Moreover, this study is situated in a relatively poor rural setting, where it is to be expected that effects associated with nutritional transition would be less evident than for the country as a whole. Few other studies examine the potential effects of pensions or other cash transfers on unhealthy consumption. One exceptional study examines the effects of a targeted cash transfer for mothers in Mexico and finds an association with excess calorie consumption [33].

There is a large body of evidence demonstrating that obesity and unhealthy consumption are leading risk factors for ill-health and disability for older people in sub-Saharan Africa [34–36]. The current focus of policy for older people in the region is heavily geared to the provision of pensions and income security [37, 38]. At the same time, older people are explicitly marginalised from the emerging global NCD agenda [39]. There is an urgent need to take a more inter-sectoral approach, including targeted public health interventions to improve awareness about nutrition and health behaviours among older people. Currently, there are almost no examples of such interventions in the region, a notable exception being a pilot project that screened older people for hypertension and provided low-sodium salt at pension pay-points [40]. The positive initial evaluation of this project points the way towards developing interventions that combine health and income security. New social pension schemes are being set up across sub-Saharan Africa. Rather than make assertions about pensions and consumption that are founded on increasingly outdated views of local context, there may be opportunities to directly link pension provision to health interventions. If this is done, then pensions may indeed provide a platform for healthy consumption and behaviours among the region’s rapidly growing older population.

## Conclusion

This study calls into question general claims about the effects of social pensions on consumption and health in low and middle-income countries. Specifically, it shows that increased consumption of food and drink associated with pension receipt does not necessarily lead to improvements in health status. There may have been links between increased consumption and enhanced health status in rural South Africa in the past when earlier studies were conducted, but these settings have experienced rapid nutritional transition and have seen sharp increases in the prevalence of obesity. Evidence from other parts of sub-Saharan Africa indicates that these changes are broadly typical of the region as a whole, albeit more advanced. As such, it cannot be assumed that providing older people with social pensions is an effective stand-alone intervention for enhancing their health.

The study also indicates a need for primary health care services to adapt to new challenges related to population ageing and non-communicable disease. At its simplest, this could take the form of more systematic provision of advice about diet, smoking and alcohol consumption. More ambitiously, there may be scope to bundle specific health promotion interventions into pension distribution systems. To date, however, there is little indication that such interventions are being developed in South Africa.

## Data Availability

The datasets generated and/or analysed during the current study are available at the Harvard Center for Population and Development Studies (HCPDS) program website: www.haalsi.org.

## Abbreviations

BMI: Body Mass Index
HAALSI: Health and Aging in Africa: a Longitudinal Study of an INDEPTH Community in South Africa
HDSS: Health and Socio-Demographic Surveillance System
NCD: Non-Communicable Disease
